# gp120 Envelope Glycoproteins of HIV-1 Group M Subtype A and Subtype B Differentially Affect Gene Expression in Human Vascular Endothelial Cells

**DOI:** 10.3390/ijms24043536

**Published:** 2023-02-10

**Authors:** Andrew J. Suh, Dante I. Suzuki, Sergiy G. Gychka, Tinatin I. Brelidze, Yuichiro J. Suzuki

**Affiliations:** 1 Department of Pharmacology and Physiology, Georgetown University Medical Center, Washington, DC 20007, USA; 2 Department of Pathological Anatomy, Bogomolets National Medical University, 01601 Kyiv, Ukraine

**Keywords:** endothelial cells, gp120, HIV, pulmonary hypertension, subtype, vascular

## Abstract

Cardiovascular complications are seen among human immunodeficiency virus (HIV)-positive individuals, who now survive longer due to successful antiretroviral therapies. Pulmonary arterial hypertension (PAH) is a fatal disease characterized by increased blood pressure in the lung circulation. The prevalence of PAH in the HIV-positive population is dramatically higher than that in the general population. While HIV-1 Group M Subtype B is the most prevalent subtype in western countries, the majority of HIV-1 infections in eastern Africa and former Soviet Union countries are caused by Subtype A. Research on vascular complications in the HIV-positive population in the context of subtype differences, however, has not been rigorous. Much of the research on HIV has focused on Subtype B, and information on the mechanisms of Subtype A is nonexistent. The lack of such knowledge results in health disparities in the development of therapeutic strategies to prevent/treat HIV complications. The present study examined the effects of HIV-1 gp120 of Subtypes A and B on human pulmonary artery endothelial cells by performing protein arrays. We found that the gene expression changes caused by gp120s of Subtypes A and B are different. Subtype A is a more potent downregulator of perostasin, matrix metalloproteinase-2, and ErbB than Subtype B, while Subtype B is more effective in downregulating monocyte chemotactic protein-2 (MCP-2), MCP-3, and thymus- and activation-regulated chemokine proteins. This is the first report of gp120 proteins affecting host cells in an HIV subtype-specific manner, opening up the possibility that complications occur differently in HIV patients throughout the world.

## 1. Introduction

The increased incidence of cardiovascular complications is seen among human immunodeficiency virus (HIV)-positive individuals, who can now survive longer because of successful antiretroviral therapies (ART). Among them, pulmonary arterial hypertension (PAH) is a serious and fatal disease characterized by increased blood pressure in lung circulation due to vasoconstriction and vascular wall remodeling, resulting in the overworking of the heart. HIV-associated PAH occurs in approximately one out of every 200 HIV-infected patients [1,2,3,4,5,6,7], which is dramatically higher than the prevalence of PAH in individuals without HIV [8]. A large prospective study of 7648 patients with HIV after the availability of potent ART showed a prevalence of right-heart catheterization-confirmed HIV-associated PAH of 0.5% (100–1000 times greater than the prevalence in individuals without HIV infection). These findings are similar to those of studies performed before the development of effective ART [5]. Thus, ART does not seem to have altered the prevalence of HIV-PAH, suggesting the direct role of HIV components in promoting PAH.

Group M Subtype B is the most prevalent subtype of HIV-1 in western countries, where most of the research data on HIV have been generated. By contrast, the majority of HIV-1 infections in eastern African and former Soviet Union countries are caused by Group M Subtype A [9,10]. However, research on the HIV-1 subtype specificity has not been rigorous, and the information available in the scientific literature may not necessarily apply to the HIV infection in many countries, possibly generating a disparity between western countries and low-income countries in developing appropriate therapeutic strategies.

The viral fusion protein of HIV-1, the envelope glycoprotein gp160, consists of a complex of gp120 and gp41 [11]. gp120 contains the binding site for the target host cell receptor and is cleaved off from gp160 by cellular proteases, including furin [12]. In the absence of the rest of the viral components, gp120 has also been shown to activate cell signaling pathways, including chemokine receptors, protein kinase C, mitogen-activated protein kinases, and reactive oxygen species in human vascular smooth muscle cells [13]. In human pulmonary artery smooth muscle cells, gp120 was found to increase intracellular calcium and induce cell growth [14]. These effects of gp120 were inhibited by an inhibitor of CCR5, a coreceptor for cellular HIV entry. In human lung microvascular endothelial cells, gp120 promoted apoptosis and the secretion of endothelin-1, which mediated the development of PAH [15].

These previous studies on the effects of vascular cells used gp120 of HIV-1 Group M Subtype B only. Despite many individuals in the world being infected with HIV-1 Group M Subtype A, information about the cellular effects of gp120 of Subtype A is completely absent. Thus, in the present study, we asked whether the gp120 proteins of HIV-1 Group M Subtype A and Subtype B may exhibit different effects on cultured human pulmonary artery endothelial cells. 

## 2. Results

### 2.1. Sequence Analysis of gp120 Subtypes A and B

**Figure 1:**
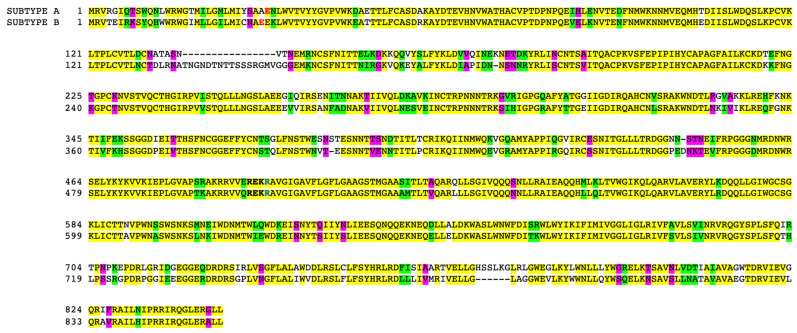
Amino acid sequence alignment of gp160 Subtype A and Subtype B. Amino acid sequence alignment of gp160 proteins of HIV-1 Group M Subtype A (Accession # AAT67478.1) and Subtype B (AAA44191.1). The furin cleavage sequence (REKR) is shown in bold. Amino acids that are identical between Subtype A and Subtype B are highlighted in yellow, and strongly and weakly similar amino acids are highlighted in green and purple, respectively, in accordance with the definition by Clustal Omega. The first and last amino acids of the recombinant pg120 proteins of Subtypes A and B that were used experimentally in this study are shown in bold red and blue letters, respectively.

We first analyzed the sequences of gp160s from HIV-1 Group M Subtypes A and B. The amino acid sequences of gp160 proteins of HIV-1 Subtype A (Accession # AAT67478.1) and Subtype B (Accession # AAA44191.1) are shown in Figure 1. The furin cleavage sequence Arg-Glu-Lys-Arg [16], where gp120 and gp41 become separated, is shown in bold. The amino acids that are identical between Subtype A and Subtype B are shown in yellow, and the strongly and weakly similar amino acids between the two proteins are shown in green and purple, respectively. We calculated that the amino acid sequences of these two proteins share 75.8% identity. The first amino acids of the recombinant gp120 proteins used in this study are shown in bold red letters, and the last amino acids in bold blue. These gp120 proteins share only 70.6% identical amino acids. Thus, we hypothesized that the two proteins may affect cells differently.

We used recombinant gp120 proteins purchased from Sino Biological. The gp120 protein of HIV-1 Group M Subtype A (isolate 92RW020, accession # AAT67478.1) consisted of amino acids Glu31-Arg494; and the gp120 protein of Subtype B (isolate BAL, accession # AAA44191.1) consisted of amino acids Glu30-Arg509. Both proteins were expressed in HEK293 cells with a polyhistidine tag at the C-terminus and lyophilized from sterile PBS at pH 7.4 (Table 1). As controls, we used SARS-CoV-2 spike protein S1 subunit (YP_009724390.1; Val16-Arg685) and SARS-CoV-2 spike protein B.1.1.529/Omicron variant S1 subunit (YP_009724390.1; Met1-Arg685), polyhistidine-tagged recombinant proteins expressed in HEK293 cells. The SARS-CoV-2 spike protein S1 subunit shares low amino acid similarity with the gp120 protein, 7.3% amino acid identity with gp120 of Subtype A, and 8.5% with Subtype B, allowing us to test the specificity of the detected responses. Both gp120 of HIV-1 and S1 of SARS-CoV-2 can be cleaved off by furin and may participate in promoting cardiovascular pathologies [17,18].

### 2.2. gp120 Subtypes A and B Differentially Affect Gene Expression

Human pulmonary artery endothelial cells were treated with recombinant gp120 of Subtypes A and B to determine how these proteins may change the gene expression patterns. We first used the R&D Proteome Profiler Array Human XL Oncology Array Kit, which allows for simultaneous monitoring of the expression of various proteins, many of which are involved in cell signaling regulation. 

After treating cells for 20 h at 1 nM of gp120 proteins or SARS-CoV-2 spike proteins, cell lysates were prepared and subjected to the protein array. Three separate experiments were performed for each group to compare Subtypes A and B; the densitometry analysis was performed for each array; and conclusions were made based on a statistical analysis of the results. An average of two spots in each array was used to determine the mean values for three arrays per group. 

**Figure 2:**
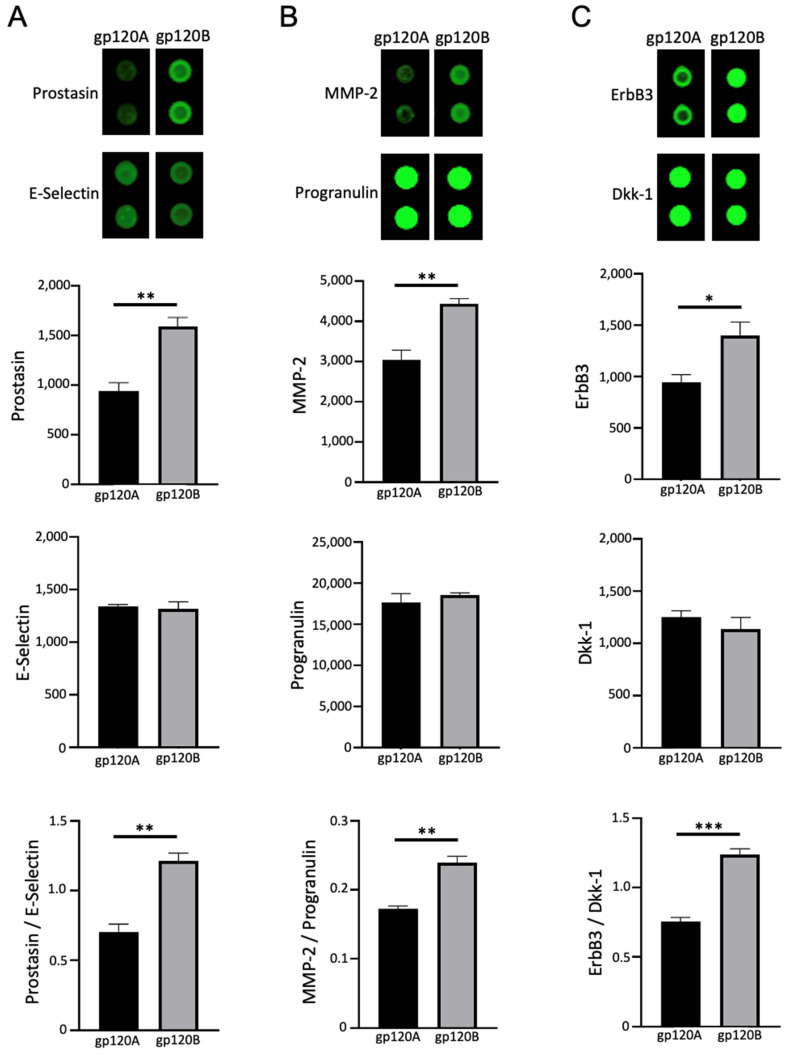
gp120 proteins of HIV-1 Group M Subtype A and Subtype B differentially affect prostasin, MMP-2, and ErbB3 expression. Human pulmonary artery endothelial cells were treated with gp120 of HIV-1 Subtype A (gp120A) or Subtype B (gp120B) at 1 nM for 20 h in triplicate. Expression patterns of various proteins were monitored using R&D Human XL Oncology Array. (**A**) Prostasin was found to be higher in gp120B-treated cells, while neighboring E-selectin spots were unchanged. (**B**) MMP-2 was found to be higher in gp120B-treated cells, while neighboring progranulin spots were unchanged. (**C**) ErbB3 was found to be higher in gp120B-treated cells, while neighboring Dkk-1 spots were unchanged. Representative images are shown at the top. Densitometry values from two spots from each array were averaged, and statistical analysis was performed using results from three separate treatments/arrays. Bar graphs represent means ± SEM (N = 3 for all groups). * *p* < 0.05. ** *p* < 0.01. *** *p* < 0.001. The *y*-axis indicates the mean pixel density of protein spots.

This approach revealed proteins that were differentially affected by HIV gp120 Subtype A and gp120 Subtype B. One protein whose expression was found to be differentially affected by Subtypes A and B is prostasin/Prss8. Figure 2A shows the representative images of prostasin protein spots in the protein arrays, demonstrating that cells treated with Subtype B gp120 have higher prostasin expression than those treated with Subtype A gp120. We also show, as a reference, neighboring spots of E-selectin that had similar intensities between Subtypes A and B. The difference between the effects of gp120 of Subtypes A and B on prostasin expression was found to be statistically significant, as indicated in the bar graph (Figure 2A). Alternatively, the expression levels of E selectin were not significantly different, and the ratio of prostasin to E selectin showed a significant difference between Subtypes A and B. 

Similarly, matrix metalloproteinase-2 (MMP-2) was found to be differentially expressed after the treatment of cells with either gp120 of Subtype A or Subtype B (Figure 2B). Alternatively, spots representing progranulin did not exhibit subtype differences, and the ratio of these proteins showed significant differences between the effects of Subtypes A and B (Figure 2B).

The expression of ErbB3 (Her3) was also found to be higher in cells treated with Subtype B gp120 compared with cells treated with Subtype A gp120 (Figure 2C). The differences in the intensities of these spots were statistically significant. Alternatively, the levels of nearby Dkk-1 spots were found to be similar between cells treated with subtypes A and B, and the normalization of ErbB3 values to Dkk-1 values showed a significant difference. 

These results revealed, for the first time, that gp120 proteins from different HIV subtypes can exhibit different effects on human host cells. 

### 2.3. gp120 Subtypes A and B Differentially Downregulates Prostasin, MMP-2, and ErbB3

**Figure 3:**
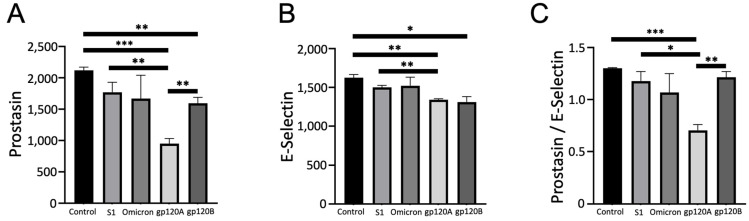
Characteristic of the effects of gp120 proteins of HIV-1 Group M Subtype A and Subtype B on the prostasin expression. Human pulmonary artery endothelial cells were treated with SARS-CoV-2 spike proteins S1, Omicron S1, and gp120 of HIV-1 Subtype A (gp120A) or Subtype B (gp120B) at 1 nM for 20 h in triplicate. Expression patterns of various proteins were monitored using R&D Human XL Oncology Array. Bar graphs represent means ± SEM (N = 3 for all groups) of (**A**) prostasin expression, (**B**) E-selectin expression, and (**C**) the ratio of prostasin to E-selectin expression. * *p* < 0.05. ** *p* < 0.01. *** *p* < 0.001. The *y*-axis indicates the mean pixel density of protein spots.

We further compared the prostasin expression levels in cells treated with gp120 proteins with untreated cells as well as spike protein-treated cells. Figure 3A shows that gp120 of Subtype A and Subtype B both caused significant downregulation of prostasin compared with untreated cells. This downregulation of prostasin was promoted significantly more potently by Subtype A gp120 compared with Subtype B. Both gp120 subtypes promoted significant downregulation of E-selectin, but not to the same extent between the subtypes (Figure 3B). The analysis of prostasin values normalized with E-selectin demonstrated that only gp120 of Subtype A, but not gp120 of Subtype B, caused a significant downregulation (Figure 3C). SARS-CoV-2 spike proteins (either the originally reported S1 subunit or the Omicron S1 subunit) did not have any significant effects on prostasin or on E-selectin expression levels, suggesting that the response for gp120 is specific (Figure 3).

**Figure 4:**
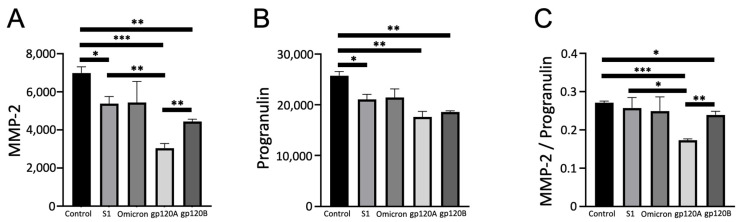
Characteristic of the effects of gp120 proteins of HIV-1 Group M Subtype A and Subtype B on the MMP-2 expression. Human pulmonary artery endothelial cells were treated with SARS-CoV-2 spike proteins S1, Omicron S1, and gp120 of HIV-1 Subtype A (gp120A) or Subtype B (gp120B) at 1 nM for 20 h in triplicate. Expression patterns of various proteins were monitored using R&D Human XL Oncology Array. Bar graphs represent means ± SEM (N = 3 for all groups) of (**A**) MMP-2 expression, (**B**) progranulin expression, and (**C**) the ratio of MMP-2 to progranulin expression. * *p* < 0.05. ** *p* < 0.01. *** *p* < 0.001. The *y*-axis indicates the mean pixel density of protein spots.

MMP-2 was also found to be significantly downregulated by both Subtypes A and B of gp120 (Figure 4A). Subtype A was significantly more potent in causing the downregulation of MMP-2. While gp120 proteins of both subtypes also caused significant downregulation of progranulin, no differences were detected in the potencies of the two subtypes (Figure 4B). The analysis of MMP-2 values normalized with progranulin showed that only Subtype A, but not Subtype B, caused a significant downregulation (Figure 4C). We also noted that the spike protein (with the original sequence) caused downregulation of MMP-2 and progranulin (Figure 4A,B). However, the downregulation effects of Subtype A gp120 were found to be more potent than those of the spike protein (Figure 4A,C). 

**Figure 5:**
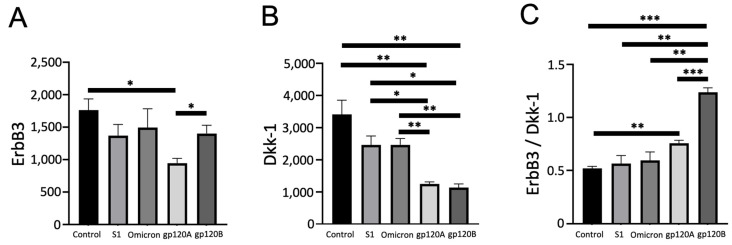
Characteristic of the effects gp120 proteins of HIV-1 Group M Subtype A and Subtype B on the ErbB3 expression. Human pulmonary artery endothelial cells were treated with SARS-CoV-2 spike proteins S1, Omicron S1, and gp120 of HIV-1 Subtype A (gp120A) or Subtype B (gp120B) at 1 nM for 20 h in triplicate. Expression patterns of various proteins were monitored using R&D Human XL Oncology Array. Bar graphs represent means ± SEM (N = 3 for all groups) of (**A**) ErbB3 expression, (**B**) Dkk-1 expression, and (**C**) the ratio of ErbB3 to Dkk-1 expression. * *p* < 0.05. ** *p* < 0.01. *** *p* < 0.001. The *y*-axis indicates the mean pixel density of protein spots.

The analysis of ErbB3 spots revealed that only gp120 of Subtype A, but not of Subtype B, caused significant downregulation of ErbB3 (Figure 5A). The neighboring protein Dkk-1, which was used as a reference in Figure 2 as a protein whose expression was not different between cells treated with either Subtype A or B gp120, was found to be significantly downregulated by both subtypes of gp120 (Figure 5B). Spike proteins did not have any effects on the expression of ErbB3 (Figure 5A). 

These results showed that gp120 of HIV Group M Subtype A potently downregulates prostasin, MMP-2, and ErbB/Her3 protein expressions in pulmonary artery endothelial cells, while gp120 of Subtype B has minimal effects.

### 2.4. gp120 Subtypes A and B Differentially Affect MCP-2 and MCP-3

**Figure 6:**
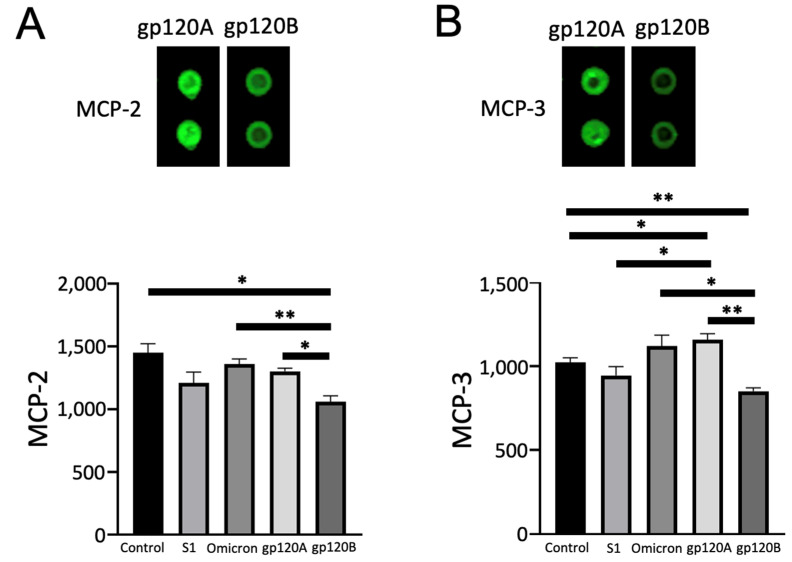
gp120 proteins of HIV-1 Group M Subtype A and Subtype B differentially affect MCP-2, and MCP-3 expression. Human pulmonary artery endothelial cells were treated with SARS-CoV-2 spike proteins S1, Omicron S1, and gp120 of HIV-1 Subtype A (gp120A) or Subtype B (gp120B) at 1 nM for 20 h in triplicate. Expression patterns of various proteins were monitored using R&D Human XL Oncology Array. (**A**) MCP-2 was found to be higher in gp120A-treated cells. (**B**) MMP-3 was found to be higher in gp120A-treated cells. Representative images are shown at the top. Densitometry values from two spots from each array were averaged, and statistical analysis was performed using results from three separate treatments/arrays. Bar graphs represent means ± SEM (N = 3 for all groups). * *p* < 0.05. ** *p* < 0.01. The *y*-axis indicates the mean pixel density of protein spots.

Conversely, we found that gp120 of Subtype B, but not Subtype A, downregulated monocyte chemotactic protein-2 (MCP-2/CCL8) and MCP-3 (CCL7). Figure 6 shows that the expression of MCP-2 and MCP-3 is higher in cells treated with gp120 Subtype A compared with cells treated with Subtype B gp120. Quantifications of these experiments determined that these differences were statistically significant. Comparisons with untreated cells revealed that Subtype A gp120 had no effect while Subtype B significantly downregulated the protein expression of MCP-2. In the case of MCP-3, we found that Subtype A gp120 significantly increases and Subtype B gp120 decreases its expression. Spike proteins did not exert any significant effects.

### 2.5. gp120 Subtypes A and B Differentially Affect TARC

**Figure 7:**
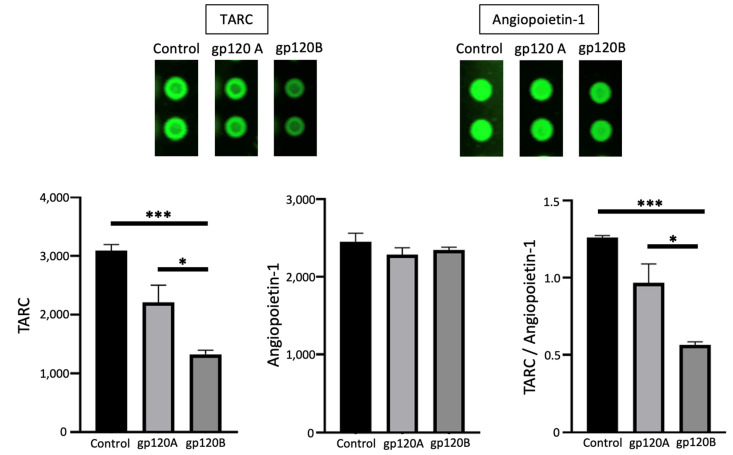
gp120 proteins of HIV-1 Group M Subtype A and Subtype B differentially affect TARC expression. Human pulmonary artery endothelial cells were treated with gp120 of HIV-1 Subtype A (gp120A) or Subtype B (gp120B) at 1 nM for 20 h. Expression patterns of various proteins were monitored using R&D Human XL Cytokine Array. TARC expression was found to be higher in gp120A-treated cells, while neighboring angiopoietin-1 spots were unchanged. Representative images are shown at the top. Densitometry values from two spots from each array were averaged, and statistical analysis was performed using results from three separate treatments/arrays. Bar graphs represent means ± SEM (N = 3 for gp120A and gp120B groups; N = 2 for untreated control). * *p* < 0.05. *** *p* < 0.001. The *y*-axis indicates the mean pixel density of protein spots.

We further performed the Human XL Cytokine Array analysis and found that thymus- and activation-regulated chemokine (TARC/CCL17) is one protein that is differentially expressed in response to gp120 of Subtypes A and B. As shown in Figure 7, the TARC expression is downregulated by gp120 of both subtypes, but Subtype B was significantly more potent than Subtype A. By contrast, in this array, angiopoietin-1 protein expression was found not to change in response to gp120 of either subtype. The value of TARC normalized to angiopoietin-1 was significantly lower in cells treated with Subtype B gp120 compared with Subtype A. 

Taken together, the gp120 proteins of HIV-1 Group M Subtypes A and B, which share ~70% homology in their amino acid sequences, differ in how they affect human host vascular endothelial cells.

## 3. Discussion

The majority of HIV-1 infections in eastern Africa and former Soviet Union countries are caused by Subtype A, while Subtype B is the most prevalent subtype in western countries [9,10]. As a consequence, much research has been carried out on Subtype B, and it is unknown whether the biological actions of the gp120 of these two subtypes differ. 

Advances in ART resulted in the long-term survival of HIV-positive individuals but also increased clinical concerns about complications that affect these individuals, such as vascular diseases, including PAH. To investigate if there could be differences in the vascular complications developed in patients infected with HIV-1 Subtype A and Subtype B, we tested the hypothesis that human host vascular cells may respond differently to the gp120 viral fusion protein of HIV-1 Group M Subtype A compared with Subtype B. In HIV, the viral fusion protein, gp120, binds to host cell receptors, particularly the CD4 of T cells, to allow the virus to enter the cell. In other cell types, recombinant HIV gp120 has been shown to activate cell signaling events [13,19,20,21,22]. 

The amino acid sequence comparison analysis of the gp120 protein of Subtype A and Subtype B revealed these proteins only share ~70% amino acid identity. The application of these proteins to cultured human pulmonary artery endothelial cells in conjunction with the use of Proteome Profiler Arrays revealed that HIV-1 Subtypes A and B elicit different protein expression changes. The major differences between the proteins consist of the absence of the TNGNDTNTTSSSRGMV sequence at positions 137–152 position in gp120 of Subunit A. Further investigations, including the role of this region, are needed to determine the molecular mechanism of the differential actions of gp120 of Subtypes A and B.

While neither clinical nor epidemiological studies have determined whether HIV-associated complications occur differently between populations affected by Subtype A and Subtype B, the results of the present molecular studies have opened up the possibility of such a difference. The major thesis of this work is to communicate to the scientific community that cells respond differently to proteins derived from the two HIV-1 subtypes. The specific proteins that were identified to be differentially expressed in cells treated with Subtype A and Subtype B gp120s have not been well studied in the context of PAH or vascular remodeling. Thus, it is premature to speculate how these protein changes may alter the clinical course of patients at this time. Our laboratory will continue investigating the actions of gp120 proteins from the two subtypes, and we expect to provide a mechanistic hypothesis on how these molecules affect patients in conjunction with the information hopefully becoming available from clinical and epidemiological studies in the future.

In summary, these results showed, for the first time, that gp120 of HIV-1 Group M Subtypes A and B exhibit different actions on human host cells, providing the basis for the need for rigorously studying the molecular differences between the actions of the HIV-1 components of different subtypes. Such efforts may offer important insights into developing therapeutic strategies to prevent and/or treat PAH and other vascular complications in HIV-positive individuals in accordance with the HIV subtypes affecting different countries. 

## 4. Materials and Methods

### 4.1. Protein Sequence Analysis

Protein amino acid sequences for gp120 of Subtype A (accession number AAT67478.1) and gp120 of Subtype B (accession number AAA44191.1) were aligned using the Clustal Omega multiple sequence alignment tool provided by the European Bioinformatics Institute, Cambridgeshire, UK [23]. The percent of identical amino acids was calculated using the equation: (number of identical amino acids)/[(number of identical amino acids) + (number of nonidentical amino acids)].

### 4.2. Cell Culture

Human pulmonary artery endothelial cells (catalog number C-12241) were purchased from PromoCell GmbH (Heidelberg, Germany). Cells were cultured in Endothelial Cell Growth Medium (PromoCell, catalog number C-22010) in accordance with the manufacturer’s instructions in 5% CO_2_ at 37 °C. Cells at passages 3–6 were placed in low-fetal bovine serum (0.4%)-containing medium before the treatment, as routinely performed in experiments on cell signaling [24]. 

Cells were treated in triplicate with either the recombinant gp120 of HIV-1 Group M Subunit A (catalog number 40403-V08H, Sino Biological, Inc., Beijing, China), gp120 of HIV-2 Group M Subunit B (catalog number 40404-V08H, Sino Biological), S1 of SARS CoV-2 spike protein (catalog number 40591-V08H, Sino Biological), or S1 of the omicron variant SARS CoV-2 spike protein (catalog number 40591-V08H41, Sino Biological). The characteristics of these recombinant proteins are summarized in Table 1.

### 4.3. Protein Array

Proteome Profiler Array Human XL Oncology Array (Catalog Number ARY026) and Human XL Cytokine Array (Catalog Number ARY022B) from R&D Systems, Inc. (Minneapolis, MN, USA) were used to identify proteins differentially affected by gp120 of HIV-1 Subtypes A and B. Cell lysates were applied to the protein arrays to detect interactions. To prepare cell lysates, cells were washed in phosphate buffered saline and solubilized with Lysis Buffer 17 (Catalog Number 895943) supplemented with leupeptin (10 µg/mL) and aprotinin (10 µg/mL) by vortexing and gentle rocking at 4 °C for 30 min. Samples were then centrifuged at 14,000× *g* for 10 min at 4 °C, supernatants were collected, and protein concentrations were determined using Bio-Rad Protein Assay (Bio-Rad, Hercules, CA, USA). 

Arrays were performed in accordance with the manufacturer’s instructions. Briefly, each membrane was incubated with Array Buffer 6 for 1 h at room temperature on a rocking platform shaker. Solutions were then replaced with appropriate Array Buffer solutions containing cell lysates (100 μg protein) and incubated overnight at 4 °C on a rocking platform shaker. Membranes were washed 3 times with Wash Buffer and incubated in the Detection Antibody solution for 1 h at room temperature on a rocking platform shaker. Membranes were again washed 3 times with Wash Buffer and incubated with IRDye 800CW Streptavidin (1:2000; LI-COR, Lincoln, NE, USA) for 30 min at room temperature. Lastly, membranes were washed 3 times with Wash Buffer, and signals were obtained by using the Odyssey Infrared Imaging System (LI-COR). Three separate arrays were performed for each group. Two spots in each array were averaged, and values for the three arrays were used to calculate means and standard errors of the mean (SEM). Since the Proteome Profiler Arrays do not contain usual housekeeping control proteins, in some calculations, the values of proteins that were unchanged in response to treatments were used for normalization. 

### 4.4. Statistical Analysis

Two groups were compared by a two-tailed Student’s *t* test, and differences between more than two groups were determined by the analysis of variance (ANOVA). *p* < 0.05 was defined as statistically significant.

## Figures and Tables

**Table 1:** Characteristics of recombinant proteins used in this study.

**Protein Name:** HIV-1 (group M, subtype A, isolate 92RW020) Envelope glycoprotein gp160 Protein (gp120 subunit).
**Catalog Number:** Sino Biological 40403-V08H.
**Protein Construction:** A DNA sequence encoding the HIV-1 envelope glycoprotein gp160 extracellular domain (Glu31-Arg494), termed gp120, was expressed with a polyhistidine tag at the C-terminus.
**Accession Number:** AAT67478.1.
**Expression Host:** HEK293 Cells.
**Molecular Mass:** 465 amino acids and predicts a molecular mass of 52.2 kDa.
**Formulation:** Lyophilized from sterile PBS, pH 7.4.
**Protein Name:** HIV-1 (group M, subtype B, isolate BAL) gp120 Protein.
**Catalog Number:** Sino Biological 40404-V08H.
**Protein Construction:** A DNA sequence encoding the HIV1 (group M, subtype B, isolate BAL) Envelope glycoprotein gp160 Protein (gp120 subunit) (Glu30-Arg509) was expressed with a polyhistidine tag at the C-terminus.
**Accession Number:** AAA44191.1.
**Expression Host:** HEK293 Cells.
**Molecular Mass:** 491 amino acids and predicts a molecular mass of 55.4 kDa.
**Formulation:** Lyophilized from sterile PBS, pH 7.4.
**Protein Name:** SARS-CoV-2 (2019-nCoV) Spike S1-His Recombinant Protein.
**Catalog Number:** Sino Biological 40591-V08H.
**Protein Construction:** A DNA sequence encoding the SARS-CoV-2 (2019-nCoV) spike protein S1 Subunit (Val16-Arg685) was expressed with a polyhistidine tag at the C-terminus.
**Accession Number:** YP_009724390.1.
**Expression Host:** HEK293 Cells.
**Molecular Mass:** 681 amino acids and predicts a molecular mass of 76.5 kDa.
**Formulation:** Lyophilized from sterile PBS, pH 7.4.
**Protein Name:** SARS-CoV-2 B.1.1.529 (Omicron) Spike S1 Protein.
**Catalog Number:** Sino Biological 40591-V08H41.
**Protein Construction:** A DNA sequence encoding the SARS-CoV-2 Spike S1 (with mutations A67V, HV69-70 deletion, T95I, G142D, VYY143-145 deletion, N211 deletion, L212I, ins214EPE, G339D, S371L, S373P, S375F, K417N, N440K, G446S, S477N, T478K, E484A, Q493R, G496S, Q498R, N501Y, Y505H, T547K, D614G, H655Y, N679K, P681H) (Met1-Arg685) was expressed with a polyhistidine tag at the C-terminus. The mutations were identified in the SARS-CoV-2 variant (known as variant B.1.1.529), which emerged in South Africa.
**Accession Number:** YP_009724390.1.
**Expression Host:** HEK293 Cells.
**Molecular Mass:** 678 amino acids and predicts a molecular mass of 76.48 kDa.
**Formulation:** Lyophilized from sterile PBS, pH 7.4.
